# Enhancement of tendon-bone interface healing and graft maturation with cylindrical titanium-web (TW) in a miniature swine anterior cruciate ligament reconstruction model: histological and collagen-based analysis

**DOI:** 10.1186/s12891-020-03199-0

**Published:** 2020-03-31

**Authors:** Keisho Ryu, Mitsuru Saito, Daisaburo Kurosaka, Seiichiro Kitasato, Toshiyuki Omori, Hiroteru Hayashi, Tomohiro Kayama, Keishi Marumo

**Affiliations:** Department of Orthopaedic Surgery, The Jikei University School of Medicine, 3-25-8, Nishi-Shimbashi, Tokyo, Minato-ku 105-8461 Japan

**Keywords:** ACL reconstruction, Enthesis, Tendon-bone interface, Titanium-web, Miniature swine, Collagen maturation, Ligamentization

## Abstract

**Background:**

Tendon-bone interface healing and ligamentization of the graft in anterior cruciate ligament **(**ACL) reconstruction with autografts are important factors affecting treatment outcome. This study aimed to investigate the effectiveness of a cylindrical titanium-web (TW) in tendon-bone interface healing and graft maturation in ACL reconstruction.

**Methods:**

Fourteen mature female CLAWN miniature swine underwent bilateral ACL reconstructions with patellar tendon (PT) autografts. In one limb, the TW/tendon complex was placed into the proximal side of the tibial tunnel. Only the graft was transplanted into the tunnel in the control limb. The proximal side of the graft was sutured into the stump of the native ACL and the distal end was stapled to the tibia. The animals were euthanized at 4 and 15 weeks postoperatively, for histological and biochemical analyses.

**Results:**

Microscopic images in TW limbs showed that ingrowth of tendon-like tissue and mineralized bone tissue into the TW connected the bone and the tendon directly. In contrast, fibrous tissue intervened between the bone and tendon in the control limbs. The total amount of collagen cross-links (which defines the strength of collagen fibers) and the maturation of collagen cross-links in TW tendons were significantly higher (*p* < 0.05) than those of control limbs. There was no significant difference in the ratio of dihydroxy-lysinonorleucine to hydroxy-lysinonorleucine (an indicator of tissue specific collagen maturation) between TW tendons and that of the native PT.

**Conclusions:**

TW promoted the maturation and formation of collagen cross-links in the grafted tendon while maintaining the cross-links pattern of native tendon collagen, and enabled direct binding of tendon to bone.

## Background

Anterior cruciate ligament **(**ACL**)** injury is one of the most common knee injuries in teenagers and young adults. Approximately 250,000 individuals, in the USA alone, suffer from ACL injury per year [1]. Usually ACL reconstruction with autografts or allografts is performed to restore function after the injury, and around 175,000 ACL reconstruction surgeries were performed in the USA annually [2]. However, delayed sports recovery and risk of re-rupture remain problematic, and many researches attempt to recreate the native ACL. Tendon-bone interface healing and ligamentization of the graft in ACL reconstruction with autografts are important factors affecting treatment outcome after surgery [3, 4]. After ACL reconstruction with a free-tendon graft, collagen fibers are first formed at the tendon-bone interface, and then collagen fibers perpendicular to the interface called the Sharpey-like fibers form to connect the tendon and bone tunnel [5, 6] . This so-called indirect insertion is different from the direct insertion found in the normal ACL tendon–bone interface or tendon–bone late healing phase at the exit of the bone tunnel [5, 7]. Direct insertions are characterized by connection of the graft and bone tunnel through the fibrocartilage tissue, forming the “tidal line” structure that stains positively for basophilia [5, 7]. Most researchers believe that indirect insertions are less effective than direct insertions and this slow and incomplete healing of the tendon-bone interface may result in inferior functional recovery and even worse osteoarthritic changes [8].

The transplanted tendon into the knee joint during ACL reconstruction undergoes a biological transformation process with changes in vasculature and collagen profiles. This remodeling process into a viable ACL-like tissue is termed “ligamentization” [4, 9]. The time required for ligamentization after ACL reconstruction has been reported to range from 9 to 18 months under light microscopy [4], 13 to 30 months under electron microscopy [4], and within 1 year in a collagen cross-links analysis [10]. The current challenge is to find a better strategy to improve tendon-bone interface healing and promote maturation of the grafted tendon for faster recovery.

Mesenchymal stem cell transplantation [11–14] and administration of biological growth factors, such as recombinant human bone morphogenetic protein-2 (rhBMP-2) [6, 14, 15], transforming growth factor (TGF) -beta1 [16], fibroblast growth factor (FGF) [5] and granulocyte colony stimulating factor (G-CSF) [17], have been performed in recent studies. Though most studies using growth factors failed to form fibrocartilage tissue at the tendon–bone interface, Hashimoto et al. reported that they succeeded in generating a tendon-bone junction similar to normal enthesis using rhBMP-2 in a rabbit model [15]. However, this method included injecting rhBMP-2 into the flexor digitorum communis tendon to induce ectopic ossicle formation, which was then surgically transferred into the tibia [15]. This method has problems in clinical application, such as the biological effects caused by growth factors and newly recruited mesenchymal stem cells, as well as high cost, and cumbersome surgical procedure.

**Fig. 1:**
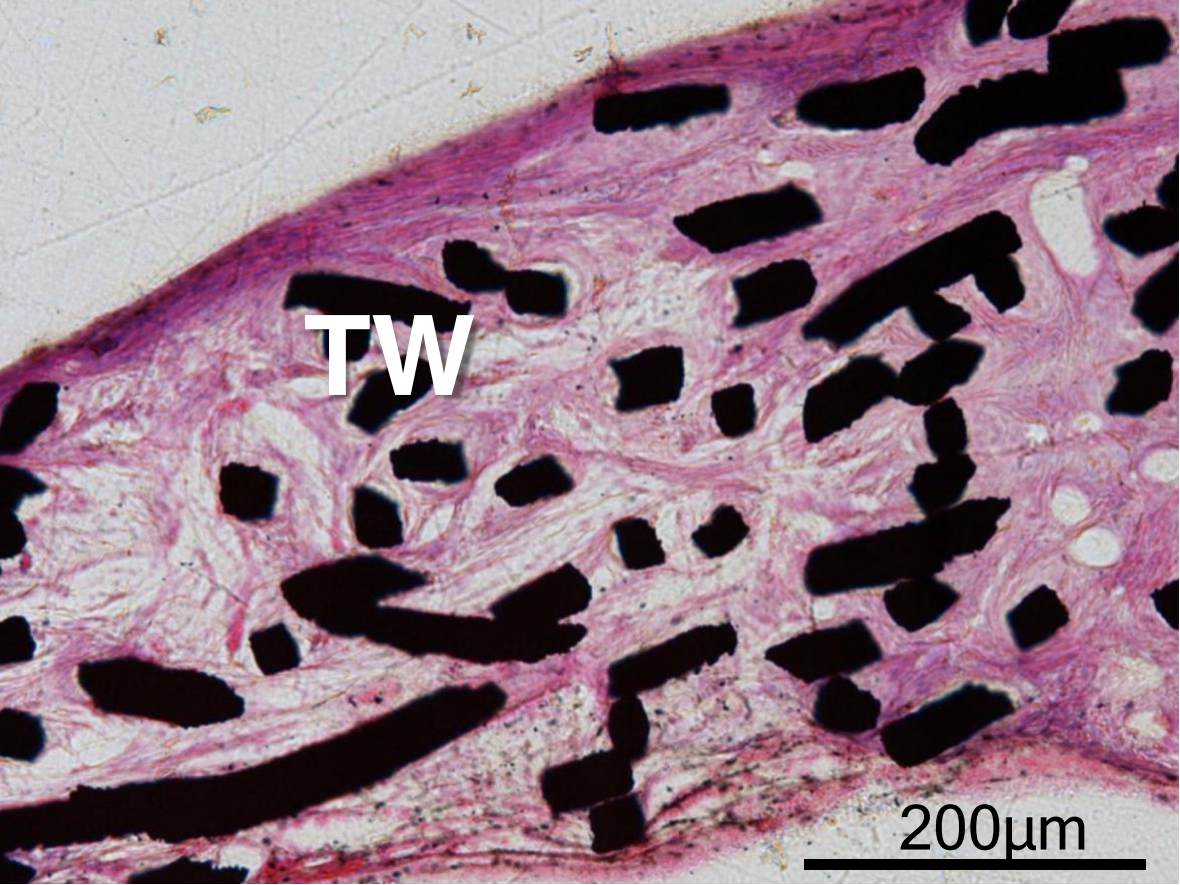
Implanted TW (titanium-web) in subcutaneous tissue of a mature female New Zealand white rabbit. Collagen synthesis growing into the TW from surrounding soft tissue was observed. Bar scale 200 μm

Strong, biodegradable materials are ideal materials for use in Orthopaedic surgeries including ACL reconstruction. However, the use of degradable screws has been limited to specific uses in ACL reconstruction and osteosynthesis. Although the advantages of bioabsorbable materials are that they eventually dissolve to eliminate the need for implant removal, and do not interfere with magnetic resonance imaging, there have been reports of increased rates of inflammation, infection and implant failure [18]. Furthermore, the clinical follow up data for degradable materials is limited compared to metal implants which have been the basis of orthopaedic procedures for many years [19]. In this study, we focused on the use of a proven strong titanium metal implant to determine whether incorporating a mesh structure may improve tendon-bone healing compared to the existing method. The titanium-web (abbreviated as TW) is an unwoven mesh composed of thin titanium fibers, whose individual cross-section is square-shaped with widths of 50–80 μm, with an inter-fibril space of 200–500 μm [20]. TW has been reported to promote bone formation and has been used as a three-dimensional scaffold for repairing bone defects in previous studies, due to its perfect biocompatibility, good intensity and excellent strength [20–22]. In our preliminary study, we implanted a TW to subcutaneous tissue and confirmed the promotion of collagen synthesis growing into the TW (Fig.1). The advantages of TW are higher biocompatibility, intensity and strength compared to degradable materials. In addition, the TW induces soft tissue ingrowth as well as bone ingrowth. TW also has the advantage of being easy to produce and process industrially, and there are fewer barriers to clinical application.

From the above preliminary experiment and previous studies, we hypothesized that transplanting the TW/tendon complex into the bone tunnel in ACL reconstruction, will promote bone and tendon-like tissue induction into the TW, thereby anchoring the tendon to bone directly. We also hypothesized that TW will have a positive effect on maturation of transplanted tendon.

The purpose of this study was to investigate the effectiveness of a cylindrical TW in tendon-bone interface healing and graft maturation in ACL reconstruction by histological and biochemical analyses. Among the available experimental animals, miniature swine were selected in this study because they are of sufficient size to perform ACL reconstructions with TW and have a weight that is closer to humans.

## Methods

### Preliminary study

Sheet TW was implanted in the subcutaneous tissue of a mature female New Zealand white rabbit. Hematoxylin–eosin staining (20x) was performed on the de-plasticized sections 10 week after surgery (Fig. 1).

### Biomaterials

**Fig. 2:**
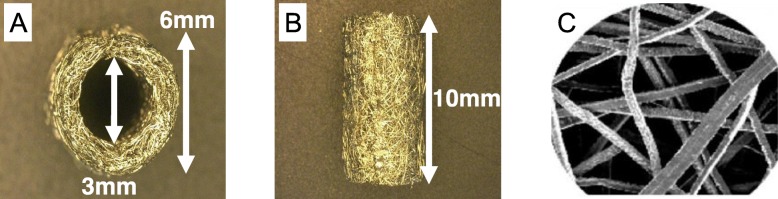
The cylindrical TW (titanium-web) consists of titanium fibers with a diameter of 50 μm. **a, b** The shape of the TW (outer diameter 6 mm, inner diameter 3 mm, length 10 mm). **c** Titanium fibers with a diameter of 50 μm

The cylindrical TW (titanium-web) with three-dimensional porous structure (outer diameter 6 mm, inner diameter 3 mm, length 10 mm) consisted of 50 μm diameter unalloyed titanium fibers (equipped to ASTM B265 Grade1), with 87% porosity and average 200 μm pore space was provided by Hi-Lex Corporation (Hyogo, Japan) for this study (Fig. 2).

### Animals

Fourteen healthy mature female CLAWN miniature swine, 30–33 months old and weighing 36 ± 10 kg were obtained from the Kagoshima Miniature Swine Research Center (Kagoshima, Japan). These animals were housed in the animal center of Hamri Co., Ltd. (Ibaraki, Japan) under an air-conditioned environment (room temperature: 20 ± 5 °C, humidity: 55 ± 15%, lighting time: 12 h a day). They were fed in pens with free access to adequate food (400–600 g pellet feed for mini-pigs per day) and water.

### Surgical procedure

**Fig. 3:**
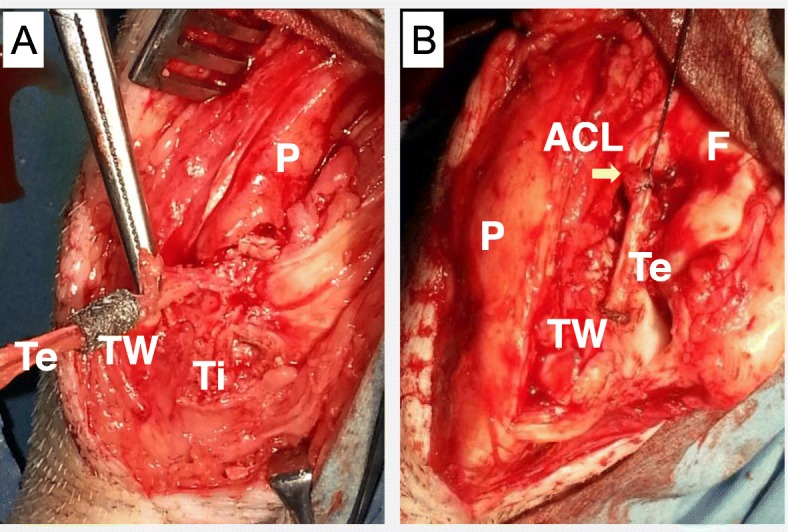
Bilateral ACL reconstruction with patellar tendon autograft. The tendon graft was inserted into the TW (titanium-web) (**a**) and the TW /tendon complex was placed into the tibial tunnel. The proximal side of the graft was sutured to the stump of the native ACL using a 4–0 nylon yarn (**b**). {TW: titanium-web, F: femur, Ti: tibia, P: patella, ACL: the stump of the native ACL (arrow), Te: the grafted tendon}

**Fig. 4:**
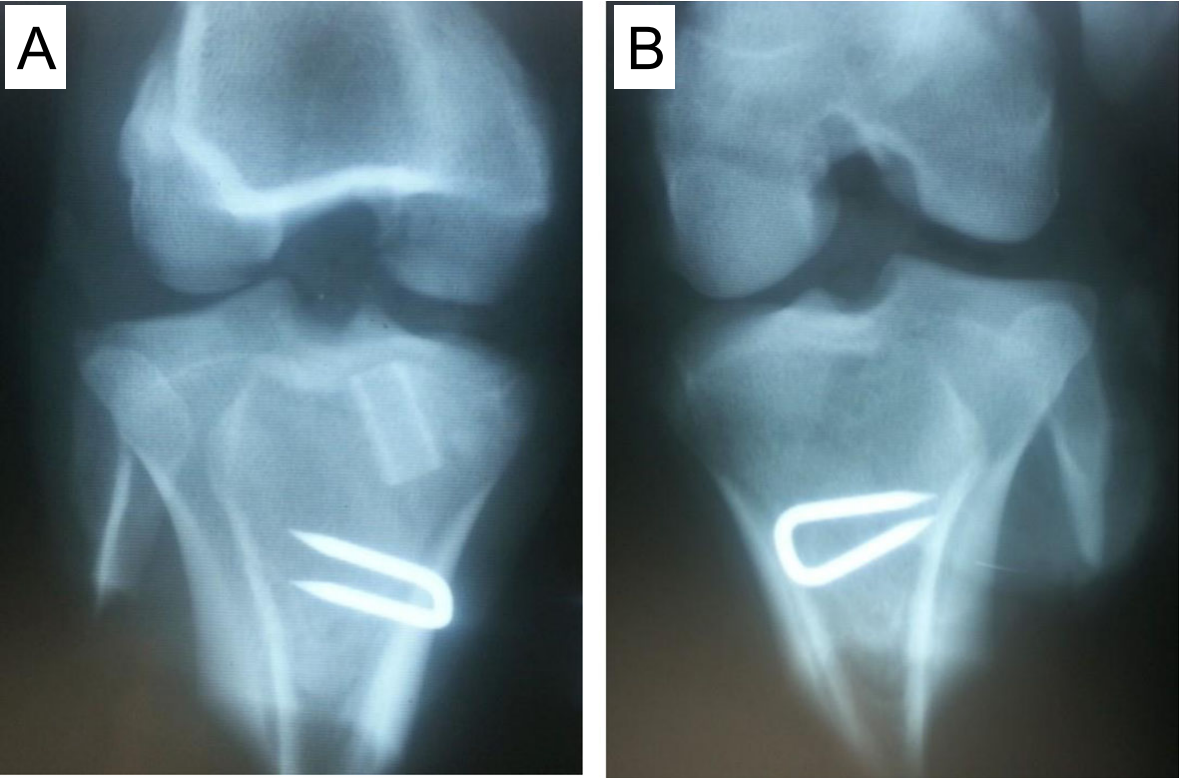
Bilateral anteroposterior radiographs of the knee after surgery. **a** TW (Titanium-web) limb. **b** Control limb

After intra-muscular administration of ketamine hydrochloride (10 mg/kg) and xylazine (4 mg/kg), each animal was fixed in a supine position on a surgical bed. Bilateral ACL reconstructions with patellar tendon (PT) autograft were carried out under the same anesthesia. An anterior longitudinal skin incision 4 cm in length and medial parapatellar arthrotomy were performed to expose the knee joint. The entire ACL was identified by removing the infra-patellar fat pad. After freeze-processing over the full-length of the ACL using liquid nitrogen, the ligament was disconnected from its tibial attachment and the ligament-bone junction of the proximal tibia was removed completely. A graft width of 3 mm for ACL reconstruction was harvested from the medial part of the patellar tendon, and disconnected at the edge of the patella. In the TW (titanium-web) limb (right knee), a 6 mm diameter tibial tunnel was created slightly inwards from the original ACL attachment. After attaching the graft inside TW (Fig. 3a), the TW/tendon complex was placed in the tibial tunnel. In the control limb, only the 3 mm graft was transplanted into the 3 mm diameter tibial tunnel. The proximal side of the graft was sutured using a 4–0 nylon yarn into the stump of the native ACL (Fig. 3b), and was fixed distally with a staple (width 5.5 mm, Daidohant, Osaka, Japan) to the tibia. The joint capsule and skin were closed with 2–0 Vicryl suture (Ethicon, Somerville, N.J., USA). No external fixation was performed and plain radiographs of the bilateral knee were taken (Fig. 4). Postoperatively, these animals were allowed to move freely in their pens. They received painkillers (20 μg/kg of Buprenorphine) and antibiotics (5 mg/kg of Enrofloxacin) for 7 days until their wound closed. Two miniature swine died on day 1 and day 18 after surgery, and the rest of animals were euthanized at 4 (*n* = 4) and 15 (*n* = 8) weeks postoperatively by overdose of pentobarbital sodium (60 mg/kg or more). All Surgical procedure were performed in the operating room of the animal center of Hamri Co., Ltd. (Ibaraki, Japan). Histological and biochemical analyses were performed by blinded observers.

### Histological analysis

At 4 and 15 weeks postoperatively (four animals from each group), the proximal end of the tibia with the transplanted graft was resected en bloc and fixed in 4% paraformaldehyde (pH 7.3), dehydrated in 70–99.5% ethanol and acetone for 2 days, exposed to a 1:1 ratio of acetone and methyl methacrylate (MMA) monomer for 3 days, further treated with MMA monomer embedded in MMA resin for 10 days, and hardened for 12 days. After polymerization, specimens were cut to a width of 200–300 μm with a microtome (BS-300CL, EXAKT, Hamburg, Germany), and ground to a thickness of 40–50 μm (MG-400CS, EXAKT, Hamburg, Germany).

Microscopic sections were de-plasticized with xylene for 2 h at 50 °C. De-plasticized sections were stained by Cole’s hematoxylin with a 1% hydrochloric acid–ethanol solution for 60 min, stained by eosin for 3 min, and washed with 99.5% ethanol (hematoxylin–eosin staining). De-plasticized sections also were stained by iron hematoxylin with a 1% hydrochloric acid–ethanol solution for 60 min, stained by Ponceau fuchsin with 1% acetic acid for 10 min, and stained with a phosphotungstic acid–phosphomolybdic acid solution. Then they were stained with naphthol green for 30 min and washed with 70–99.5% ethanol (Villanueva’s Goldner staining). In addition, Toluidine blue staining was performed on the de-plasticized sections 15 week after surgery. These stained sections were enclosed and observed with a light microscope (BX53, Olympus, Tokyo, Japan).

### Biochemical analysis

Specimens were collected from the transplanted grafts and the lateral part of the patellar tendon in both limbs of miniature swine 15 weeks postoperatively (*n* = 4). In addition to this, 2 more samples were salvaged from the 15 weeks histology swine, to make up 6 samples each for biochemical analysis.

Quantitative analysis of divalent immature and trivalent mature collagen cross-links was performed by high-performance liquid chromatography (HPLC) using a fluorescence detection method established previously [23]. Each tissue was minced and suspended in 500 vol (v/wt) of 0.05 M potassium phosphate buffer, pH 7.6 (ionic strength = 0.15 M) at 4 °C and continuously stirred for 72 h under a vacuum. A 1/30 volume of sodium borohydride (NaBH4) was added to the solution. The reaction was allowed to proceed for 60 min at 37 °C and was terminated by the addition of 6 N hydrochloric acid to decrease the pH value to 4.0. The solution residue was collected by centrifugation (3000 g for 15 min), washed twice with deionized water, and lyophilized. For HPLC analysis, lyophilized samples were dissolved in 0.2 N sodium citrate buffer (pH 2.2) and filtered through a 0.45-μm filter (Gelman Science Japan Ltd., Tokyo, Japan).

The immature cross-links, such as dihydroxy-lysinonorleucine (DHLNL) and hydroxy- lysinonorleucine (HLNL) were detected by online post column derivatization using *O*-phthalaldehyde and were simultaneously monitored by fluorescence with excitation at 350 nm and emission at 450 nm. Mature pyridinium cross-links, such as pyridinoline (PYD) and deoxypyridinoline (DPD) were detected by natural fluorescence with excitation at 295 nm and emission at 395 nm. The content of each cross-link was expressed as mol/mol of collagen. The amount of collagen in the tissue was calculated by hydroxyproline assay using an elution procedure. The ratio of DHLNL/HLNL was assessed as a surrogate marker of the degree of hydroxylation of lysine in the collagen molecules. This ratio is highly regulated and distinctive for different collagenous tissues.

### Statistical analysis

All values are listed as means with their standard deviations (mean ± SD) in the text and the tables. Statistical significance was determined by one-way analysis of variance (ANOVA) with post hoc adjustment for multiple comparisons and was established at a *p* value < 0.05. All statistical analyses were performed using statistical analysis software (JMP V.3.1.6, SAS Institute, Cary, NC).

## Results

### Drop-outs and complications

Two miniature swine died on day 1 and day 18 after surgery. The precise causes for their deaths are unknown, but there were no apparent signs of infection observed on necropsy. Lameness lasted one to 2 weeks after surgery. In one case of 15 weeks after surgery, the inserted TW/tendon complex could not be found in the tibia.

### Histological findings

#### 4 weeks after surgery (*n* = 4)

**Fig. 5:**
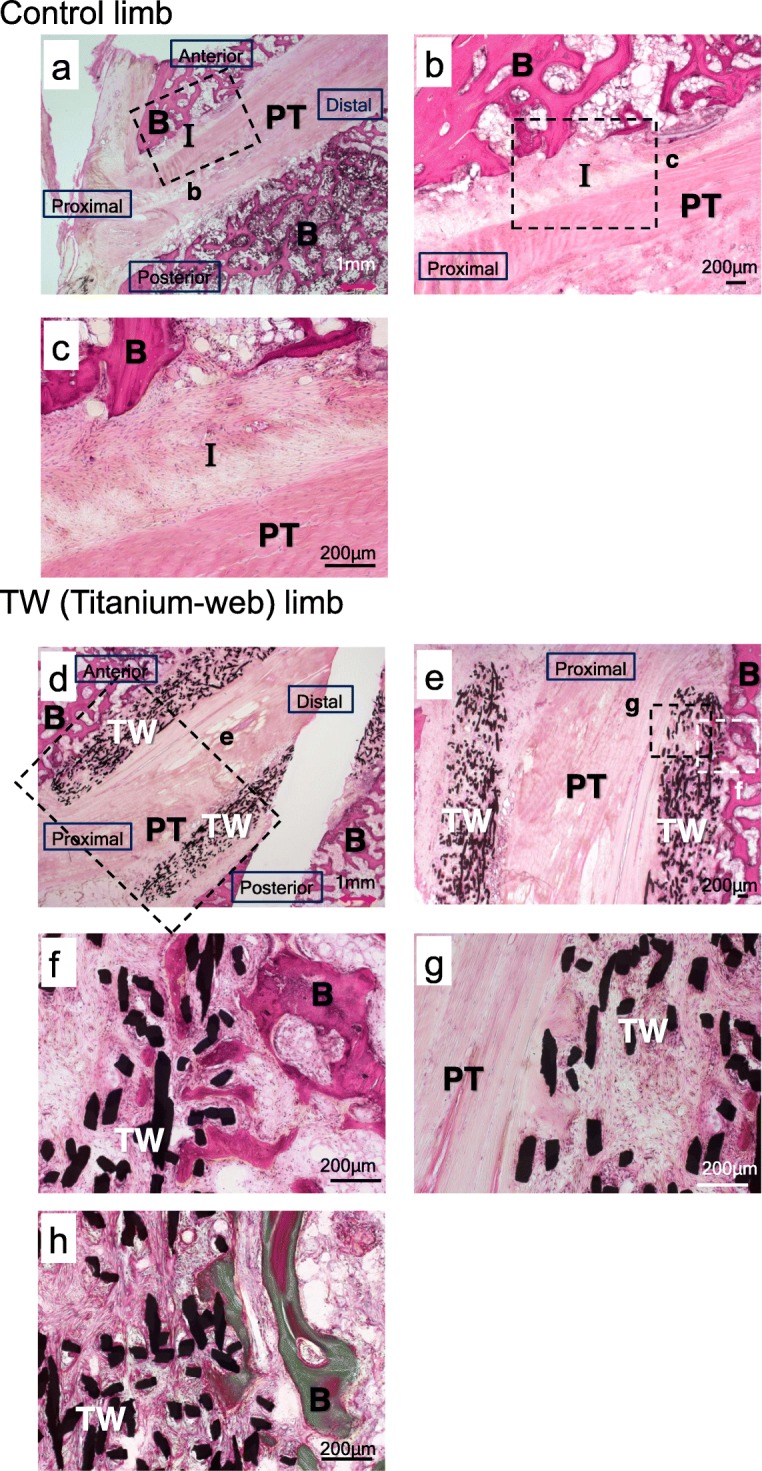
Histological examinations in control limbs (**a-c**) and TW (titanium-web) limbs (**d-h**) 4 weeks after surgery. **a** Overview picture of the control limb (H.E. staining, 0.63x). The interface tissue was observed between the tendon and the anterior side bone. **b** Low-magnification (4x) view of the boxed area in the column of (**a**). **c** High-magnification (10x) view of the boxed area in the column of (**b**). Fibrous tissue and inflammatory cells were confirmed in the interface between the tendon and bone. **d** Overview picture of the TW limb (H.E. staining, 0.63x). Tendon and bone ingrowth were observed in anterior proximal part of the TW. **e** Low magnification (2x) view of the boxed area in the column of **(d). f. g** High-magnification (10x) view of the boxed area in the column of **(e)**. Ingrowth of fibrous tissue and bone tissue in the TW was observed. **h** Villanueva’s Goldner staining. Calcified bone formation was confirmed in TW (10x). B: bone. I: interface tissue. PT: patellar tendon. TW: titanium-web. Bar scale 200 μm

In the control limbs the interface between the tendon and the anterior side bone was filled fibrous tissue and inflammatory cells. In contrast, ingrowth of fibrous tissue (18–22% of the TW graft) and bone tissue (10–20% of the TW graft) in proximal part of the TW (titanium-web) was found in experimental limbs. Villanueva’s Goldner stain showed that calcified bone formation was observed in 3 out of 4 samples (Fig. 5).

#### 15 weeks after surgery (*n* = 4)

**Fig. 6:**
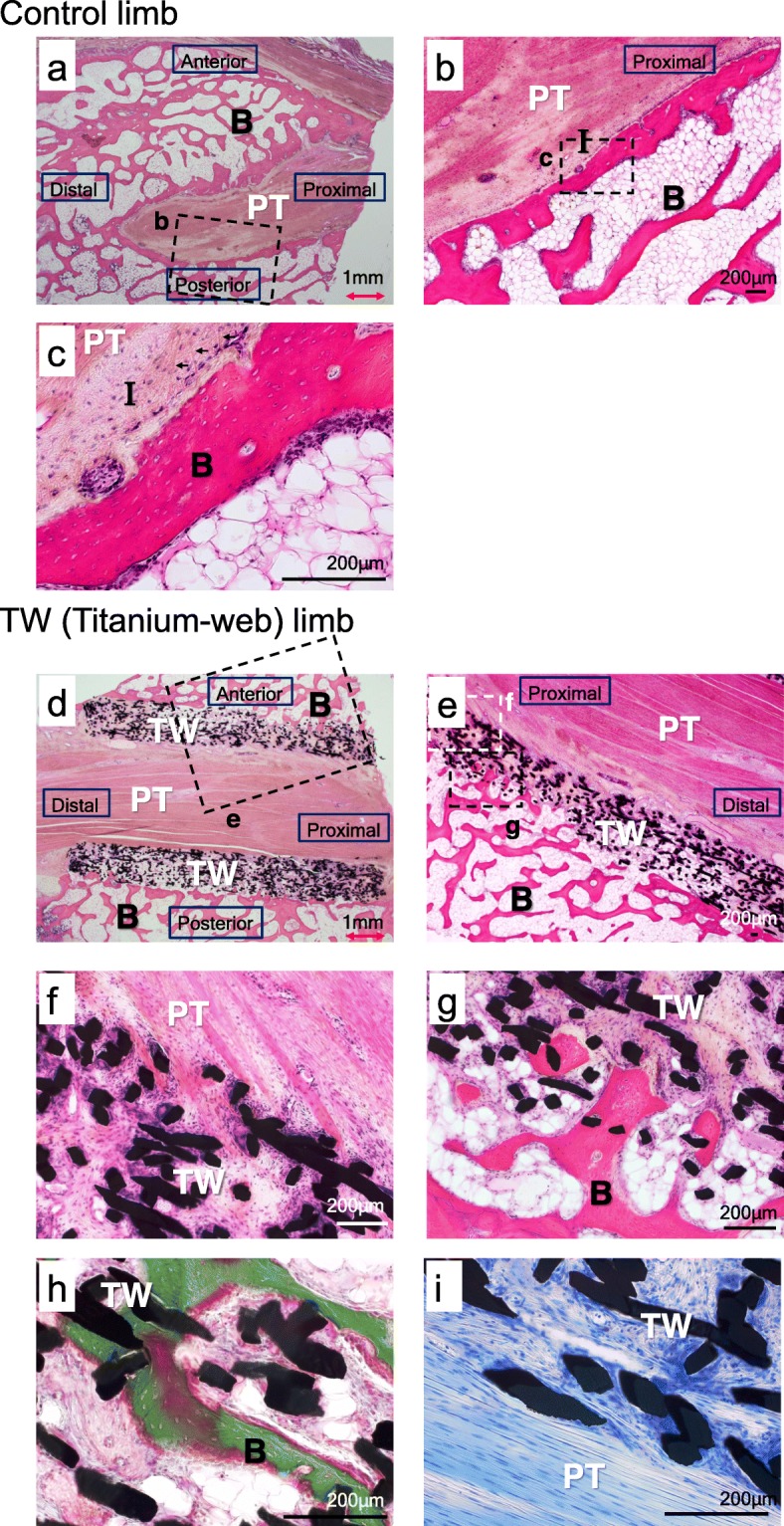
Histological examination in control limbs (**a**, **b, c**) and TW (titanium-web) limbs (**d-i**) 15 weeks after surgery. **a** Overview picture of the control limb (H.E. staining, 0.63x). The interface tissue was observed around the tendon. **b** Low-magnification (4x) view of the boxed area in the column of (**a**). The interface tissue between the tendon and the bone was more mature than at 4 weeks. **c** High-magnification (20x) view of the boxed area in the column of (**b**). Several Sharpey-like fibers (arrows) were observed in the interface layer. **d** Overview picture of the TW limb (H.E. staining, 0.63x). Although the mean tendon and bone ingrowth were greater on the anterior side, there were no significant differences between bone and tendon ingrowth in anterior and posterior sides of the TW. **e** Low magnification view (1.25x) of the boxed area in the column of (**d**)**. f** High-magnification (10x) view of the boxed area in the column of (**e**). Ingrowth of tendon and tendon-like soft tissue into the TW was more prominent than at 4 weeks. **g** High-magnification (10x) view of the boxed area in the column of (**e**). Tendon-like soft tissue and bone tissue in TW connected directly. **h** Villanueva’s Goldner staining. Calcified bone formation was confirmed in TW (20x). **i** Toluidine blue staining. Fibrocartilage tissue was not observed (20x). B: bone. I: interface tissue. PT: patellar tendon. TW: titanium-web. Bar scale 200 μm

In the control limbs the interface between tendon and bone was more expanded and mature than those at 4 weeks, and several Sharpey-like fibers were observed in the interface layer. In contrast, in the TW limbs, ingrowth of tendon-like soft tissue (38–50% of TW graft) and mineralized bone tissue (17–25% of TW graft) was observed in both side of the TW. Especially in anterior proximal part of the TW, the bone tunnel and the transplanted tendon were connected directly, without intervening tissues. No chondrocytes were observed (Fig. 6). In one of four cases, TW could not be found in the TW limb, and this case was excluded from consideration.

The raw data of the bone ingrowth rate and tendon ingrowth rate into TW is listed in additional files.

### Biochemical findings (*n* = 6)

**Fig. 7:**
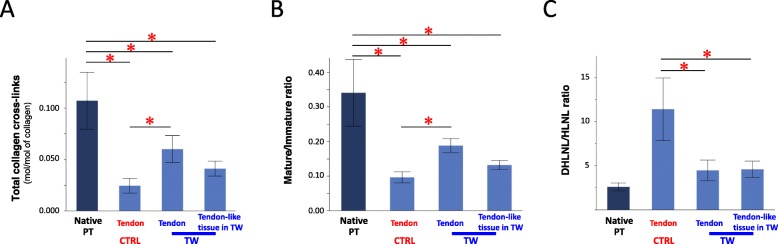
Quantitative analysis of collagen cross-links. (Native PT: native patellar tendon, CTRL: control limb, TW: titanium-web limb). **a** Total amount of collagen cross-links. **b** Maturation of collagen cross-links (the ratio of mature to immature cross-links). **c** The DHLNL/HLNL ratio in the matrices. *p < 0.05

The total number of collagen cross-links (the sum of DHLNL, HLNL, PYD) of the transplanted tendon in the TW limbs (0.060 ± 0.013 mol/mol of collagen) was, although low compared with that of the original patellar tendon (0.107 ± 0.027 mol/mol of collagen), significantly greater than that of the transplanted tendon in the control limbs (0.024 ± 0.007 mol/mol of collagen). In addition, the total number of collagen cross-links in the tendon-like tissue within the TW itself (0.041 ± 0.007 mol/mol of collagen) was found to be equivalent to that of the transplanted tendon parenchyma in the TW limbs (Fig. 7a). DPD was not detected in the tendons.

The maturation of collagen cross-links (the ratio of mature to immature cross-links) of the tendon in TW limbs (0.19 ± 0.02) also was less than that of the native PT (0.34 ± 0.10), but were significantly higher (*p* < 0.05) than that of the tendon in control limbs (0.09 ± 0.02). The maturation of collagen cross-links in the tendon-like tissue within TW itself (0.13 ± 0.01) was also found to be equivalent to that of the transplanted tendon parenchyma in the TW limbs (Fig. 7b).

The DHLNL/HLNL ratio, which is an indicator of tissue specific collagen maturation, was significantly higher in control limb tendons (11.4 ± 3.5) than in native PT (2.6 ± 0.4). On the other hand, there was no significant difference in the DHLNL/HLNL ratio between the tendon in TW limbs (4.5 ± 1.2), the tendon-like tissue in the TW (4.6 ± 0.9) and the native PT (Fig. 7c).

## Discussion

The present study demonstrated that the use of TW (titanium-web) in ACL reconstruction surgery promoted formation and maturation of collagen cross-links in the grafted tendon, and also directly binding the bone and tendon tissue through the TW.

Rodeo et al. [24, 25] reported that tendon healing occurred by initial bone ingrowth into the fibrovascular interface tissue in a dog model. There was progressive mineralization of the interface tissue, with subsequent bone ingrowth into the outer tendon and incorporation of the tendon graft into surrounding bone. As fibrovascular organization matured, the collagen fibers that attached the tendon to bone resembled Sharpey fibers. Biomechanical testing demonstrated a progressive increase in tendon pull-out strength that correlated with the amount of bone ingrowth, mineralization, and maturation of the healing tissue over 12 weeks. Similarly, on the control limbs of this study, the interface tissue between the bone and the tendon was formed 4 weeks after surgery, and the formation of Sharpey-like fibers was observed 15 weeks after surgery suggesting indirect insertion. Other hand, on the TW limbs of this experiment, ingrowth of tendon-like soft tissue was found to be similar to the native tendon tissue by collagen analysis. Furthermore, calcified bone tissue began to form in the TW 4 weeks after surgery, and connected the bone and the transplanted tendon directly within 15 weeks after surgery. Although this insertion is different from direct insertions which consists of a fibrocartilage layer, it is histologically closer to the normal insertion compared to indirect insertions.

Process of “ligamentization” was proposed by Amiel et al in 1986 [26]. Both tendons and ligaments are composed of connective tissue primarily containing types I and III collagen, proteoglycans, and cells. However, their precise composition and arrangement of matrix macromolecules differ to provide the specific mechanical properties required [27]. For example, compared with tendons, ligaments contain cells with rounded nuclei, and have more type III collagen and proteoglycans, less total collagen, different collagen cross-link composition, and different distribution of collagen fibril diameters [27]. Amiel et al demonstrated that a patellar tendon transplanted into the rabbit knee to replace an excised ACL continuously developed into a substance similar to a normal ACL [26]. They reported that spindle-shaped fibroblasts and coarse fibrillar crimp in PT autografts converted to rounded fibroblasts and fine fibrillar crimp similar to the normal ACL under histological examination. Also, collagen cross-links analysis revealed that the tissue changed from the low DHLNL PT pattern to the high DHLNL pattern observed in normal ACL [26].

Collagen cross-links of collagen fibers in tendon, ligament and bone can be divided into two types: lysine hydroxylase and lysyl oxidase-controlled cross-links (enzymatic cross-links) and glycation or oxidation-induced advanced glycation end products (AGEs) cross-links [28]. Among collagen cross-links, enzyme-dependent collagen cross-links play an important role in the strength of collagen fibers and acts as a scaffold for cell differentiation [10, 23, 28–31]. The total number of enzyme-dependent cross-links (immature cross-links + mature cross-links) is controlled by lysyl oxidase and will not be formed to exceed its required biomechanical strength [10, 28, 30, 31]. While it is difficult to directly assess the required strength of the transplanted tendons, it has been shown that the total number of collagen cross-links is indicative of collagen strength, and is significantly reduced in the transplanted tendons. The total collagen cross-links in TW tendon was greater compared to the control group tendon (Fig. 7a) to suggest a superior collagen strength.

The study also investigated for collagen maturation. Three reducible immature cross-links (dihydroxy-lysinonorleucine: DHLNL, hydroxy-lysinonorleucine: HLNL, lysinonorleucine: LNL) and two mature cross-links (pyridinoline: PYD, deoxypyridinoline: DPD) have been identified by the difference of lysine hydroxylation rate. The component ratio of the five enzyme dependent cross-links (cross-links pattern) is considered to be tissue-specific. Skin, tendon, ligament, and bone types were identified by cross-link patterns using the ratio of high hydroxide to low hydroxide cross-links (DHLNL/HLNL ratio or PYD/DPD ratio) [10, 23, 28–31]. The maturation of collagen cross-links (the ratio of mature to immature cross-links) of the transplanted tendon was significantly greater than that of the transplanted tendon in the control limbs (Fig. 7b). These results suggested that the use of TW can promote the maturation and formation of collagen cross-links in the tendon 15 weeks after ACL reconstruction.

DHLNL/HLNL ratio is also a marker for the ligamentization process of the transplanted tendon. Collagen cross-links analysis in the autologous tendon implanted in the human joint is initially subjected to a temporary lack of blood flow, reducing cellular synthesis of collagen. Thereafter, blood circulation is restored, promoting collagen synthesis, leading to collagen content increase, which results in increased immature cross-links and collagen fiber formation. As mechanical load is applied, immature cross-links are converted to mature cross-links. Finally, collagen cross-links pattern, converted from the tendon type (low DHLNL/HLNL ratio) seen in semitendinosus, gracilis and patellar tendons into the ligament type (high DHLNL/HLNL ratio) in the native ACL, is observed within 1 year postoperatively [10]. In this study, early to middle stage ligamentization was analyzed. The DHLNL/HLNL ratio in the tendon parenchyma of control limbs increased significantly. In contrast, the ratio in the tendon parenchyma and tendon-like tissue in the TW maintained its tendon type pattern, similar to that of the original patellar tendon (Fig. 7c). This result suggested that the use of TW does not adversely affect the collagen pattern, and also promotes a cross-link pattern similar to the native tendon. In order to assess the late stage of ligamentization in the transplanted tendon, DHLNL/HLNL ratio needs to be measured at a later stage of tendon maturation.

On the other hand, possible complications include TW deformation, breakage, and displacement. Since the TW is composed of thin titanium fibers, applying a strong force may result in deformation, and special attention was required when inserting the graft into the TW. However, breakage of TW was not observed in this study or in previous studies. Displacement of the TW was confirmed in one case in this study. On close examination, not only was the TW displaced completely from the bone tunnel, but the transplanted tendon itself could not be identified. These findings suggest that the fixation of the tendon to the tibia was inadequate, resulting in displacement of both the TW and the tendon from the tibial bone tunnel. This may be a problem encountered only in animal experiments, as in clinical practice, the use of ACL brace and controlled rehabilitation limit excessive movement after surgery.

Patellar tendon was used in this study as they are often selected as a graft clinically. Its cellular properties vary depending on the mechanical force that they encounter [32, 33]. Tenocytes play an important role in the synthesis of collagen and proteoglycans and thus maintenance of the surrounding extracellular matrix [34], Among tenocytes are also multipotent precursor cells to aid remodeling and adaptation to different surroundings [35]. These tendon properties, together with mechanical stimulation, are important characteristics for a graft that undergoes transformation, and thus contributing to ligamentization. It is also important to note that the swine were allowed to move freely post-operatively. This preserves mechanical stimulation of the transplanted grafts, which prevents collagen fiber atrophy and graft failure. Further studies are warranted to evaluate the different strains on the tendon grafts during movement, and also to determine the optimum rehabilitation to promote tendon ingrowth into the TW.

There are some limitations to this study that must be addressed. First, the exact mechanism that promotes the formation of bone tissue and the maturation of the tendon tissue by the TW is still unclear, and warrants further consideration. Secondly, the formation of a fibrocartilaginous layer was not seen during the observed period. It is possible that a more natural structural regeneration of the tendon-bone junction may occur with more time. Adequate mechanical stresses and strong fixation are required to regenerate a firm tendon-bone junction, and it is possible that a stronger connection may be achieved by altering the shape and position of the TW. Thirdly, we did not perform biomechanical tests in the present study due to the limited number of samples. Further studies are needed not only to determine the breaking load and stiffness but to analyze the diameter and size of TW. Lastly, the procedure requires a relatively large bone tunnel compared to the graft tendon alone, because it is necessary to pass the transplanted tendon with the TW complex through the tunnel. When considering clinical application, damage to the meniscus or the transverse ligament can occur when porting a clinically sufficient thickness of the tendon, or bone tunnels can overlap in two-root reconstruction. These problems may be solved by improving the installation position of the TW.

## Conclusions

We investigated the effectiveness of cylindrical TW (titanium-web) in tendon-bone interface healing and graft maturation in a miniature swine ACL reconstruction model. The present study suggested the following: (1) TW promoted ingrowth of tendon-like soft tissue and calcified bone tissue into TW connecting the bone and transplanted tendon directly within 15 weeks after surgery. (2) TW promoted the maturation and the formation of collagen cross-link in the tendon while maintaining the cross-links pattern of collagen at 15 weeks after surgery.

Despite further studies such as biomechanical test, biochemical test comparing to the native ACL are needed, our method using a clinically safe titanium material in a single surgery has potential clinical applications not only for ACL reconstruction, but also in cases in which the tendon insertion site has been destroyed by a tumor, or for tendon ruptures around the tendon-bone junction.

## Supplementary information

**Additional file 1.** The raw data of the bone ingrowth rate and tendon ingrowth rate into TW.

**Additional file 2.** The raw data and the descriptive statistics data of collagen cross-links analysis.

## Data Availability

The datasets used and/or analyzed during the current study are available from the corresponding author on reasonable request. All data generated or analyzed during this study are included in this published article and its Additional files 1 and 2.

## Abbreviations

ACL: Anterior cruciate ligament
AGEs: Advanced glycation end products
ANOVA: One-way analysis of variance
DHLNL: Dihydroxy-lysinonorleucine
DPD: Deoxypyridinoline
HLNL: Hydroxy- lysinonorleucine
HPLC: High-performance liquid chromatography
LNL: Lysinonorleucine
MMA: Methyl methacrylate
PT: Patellar tendon
PYD: Pyridinoline
rhBMP-2: Recombinant human bone morphogenetic protein-2
TW: Titanium-web
