# Supplementary material for: Enhancement of tendon-bone interface healing and graft maturation with cylindrical titanium-web (TW) in a miniature swine anterior cruciate ligament reconstruction model: histological and collagen-based analysis
Source: BMC Musculoskelet Disord. 2020 Mar 31;21:198. doi: 10.1186/s12891-020-03199-0 (PMC7110724; doi:10.1186/s12891-020-03199-0)
Supplement: Supplementary file 1 — Additional file 1. The raw data of the bone ingrowth rate and tendon ingrowth rate into TW. [file 12891_2020_3199_MOESM1_ESM.docx]

Maximum bone and tendon ingrowth rates into the TW

Ingrowth rate = Ingrowth depth / Titanium thickness 3mm

|  | Anterior side bone ingrowth | Anterior side tendon ingrowth | Posterior side bone ingrowth | Posterior side tendon ingrowth |
| --- | --- | --- | --- | --- |
| 4w - SJ31 | 0.2 | 0.13 | 0.17 | 0.22 |
| 4w - SJ48 | 0.11 | 0.14 | 0.05 | 0.21 |
| 4w - SK74 | 0.29 | 0.24 | 0 | 0.25 |
| 4w - SK12 | 0.2 | 0.19 | 0.19 | 0.18 |
| 4w - mean | 0.2 | 0.18 | 0.1 | 0.22 |
|  |  |  |  |  |
|  | Anterior side bone ingrowth | Anterior side tendon ingrowth | Posterior side bone ingrowth | Posterior side tendon ingrowth |
| 15W - SJ02 | 0.25 | 0.35 | 0 | 0.32 |
| 15W - SJ66 | 0.3 | 0.7 | 0.29 | 0.33 |
| 15W - SK10 | 0.2 | 0.46 | 0.22 | 0.49 |
| 15w - mean | 0.25 | 0.5 | 0.17 | 0.38 |
