# Supplementary material for: Enhancement of tendon-bone interface healing and graft maturation with cylindrical titanium-web (TW) in a miniature swine anterior cruciate ligament reconstruction model: histological and collagen-based analysis
Source: BMC Musculoskelet Disord. 2020 Mar 31;21:198. doi: 10.1186/s12891-020-03199-0 (PMC7110724; doi:10.1186/s12891-020-03199-0)
Supplement: Supplementary file 2 — Additional file 2. The raw data and the descriptive statistics data of collagen cross-links analysis. [file 12891_2020_3199_MOESM2_ESM.docx]

Raw data of collagen analysis

|  | DHLNL | HLNL | PYD | Total  crosslinks | D/H | Maturation |
| --- | --- | --- | --- | --- | --- | --- |
| 0 Native No.1 | 0.0429 | 0.0184 | 0.0234 | 0.0847 | 2.3315 | 0.3817 |
| 0 Native No.2 | 0.0621 | 0.0195 | 0.0244 | 0.106 | 3.1846 | 0.299 |
| 0 Native No.3 | 0.0585 | 0.0201 | 0.0192 | 0.0977 | 2.9104 | 0.243 |
| 0 Native No.4 | 0.0791 | 0.0289 | 0.0540 | 0.1621 | 2.737 | 0.5009 |
| 0 Native No.5 | 0.0487 | 0.0205 | 0.0250 | 0.0943 | 2.3756 | 0.3627 |
| 0 Native No.6 | 0.0517 | 0.0263 | 0.0199 | 0.0978 | 1.9658 | 0.2538 |
|  |  |  |  |  |  |  |
|  | DHLNL | HLNL | PYD | Total  crosslinks | D/H | Maturation |
| 1 CTRL No.1 | 0.0231 | 0.0024 | 0.0029 | 0.0284 | 9.625 | 0.1145 |
| 1 CTRL No.2 | 0.0113 | 0.0013 | 0.0012 | 0.0138 | 8.6923 | 0.096 |
| 1 CTRL No.3 | 0.0291 | 0.0016 | 0.0033 | 0.034 | 18.1875 | 0.1081 |
| 1 CTRL No.4 | 0.0231 | 0.0019 | 0.0022 | 0.0272 | 12.1579 | 0.0876 |
| 1 CTRL No.5 | 0.0159 | 0.0016 | 0.0012 | 0.0187 | 9.9375 | 0.0697 |
| 1 CTRL No.6 | 0.0201 | 0.0021 | 0.0022 | 0.0244 | 9.5714 | 0.0995 |
|  |  |  |  |  |  |  |
|  | DHLNL | HLNL | PYD | Total  crosslinks | D/H | Maturation |
| 2 TW tendon No.1 | 0.0358 | 0.0059 | 0.0092 | 0.0508 | 6.0816 | 0.2202 |
| 2 TW tendon No.2 | 0.0478 | 0.0094 | 0.0101 | 0.0672 | 5.1026 | 0.1771 |
| 2 TW tendon No.3 | 0.0358 | 0.0083 | 0.0088 | 0.0528 | 4.3188 | 0.1986 |
| 2 TW tendon No.4 | 0.0589 | 0.0121 | 0.0114 | 0.0825 | 4.8614 | 0.1606 |
| 2 TW tendon No.5 | 0.0395 | 0.0109 | 0.0091 | 0.0595 | 3.6154 | 0.1807 |
| 2 TW tendon No.6 | 0.0294 | 0.0107 | 0.0076 | 0.0477 | 2.7528 | 0.1892 |
|  |  |  |  |  |  |  |
|  | DHLNL | HLNL | PYD | Total  crosslinks | D/H | Maturation |
| 3 TW tendon-like No.1 | 0.0287 | 0.0053 | 0.0052 | 0.0392 | 5.3902 | 0.1523 |
| 3 TW tendon-like No.2 | 0.0276 | 0.006 | 0.0039 | 0.0374 | 4.6087 | 0.1155 |
| 3 TW tendon-like No.3 | 0.0285 | 0.0051 | 0.0046 | 0.0381 | 5.6154 | 0.1372 |
| 3 TW tendon-like No.4 | 0.0387 | 0.0079 | 0.0059 | 0.0525 | 4.8852 | 0.1256 |
| 3 TW tendon-like No.5 | 0.0326 | 0.009 | 0.0051 | 0.0467 | 3.6377 | 0.1222 |
| 3 TW tendon-like No.6 | 0.0218 | 0.0066 | 0.0039 | 0.0324 | 3.2941 | 0.1365 |

Descriptive statistics data

| 0 Native |  |  |  |  |  |  |
| --- | --- | --- | --- | --- | --- | --- |
|  | DHLNL | HLNL | PYD | Total  crosslinks | D/H | Maturation |
| Count | 6 | 6 | 6 | 6 | 6 | 6 |
| Mean | 0.057167 | 0.022283 | 0.02765 | 0.1071 | 2.58415 | 0.340183 |
| Unbiased Variance | 0.000162 | 1.81E-05 | 0.000172 | 0.000773 | 0.196005 | 0.009333 |
| Standard deviation | 0.012746 | 0.004259 | 0.013126 | 0.027809 | 0.442724 | 0.096606 |
| Standard error | 0.005204 | 0.001739 | 0.005359 | 0.011353 | 0.180741 | 0.039439 |
| Minimum | 0.0429 | 0.0184 | 0.0192 | 0.0847 | 1.9658 | 0.243 |
| Maximum | 0.0791 | 0.0289 | 0.054 | 0.1621 | 3.1846 | 0.5009 |
| Coefficient of variation | 0.222961 | 0.191122 | 0.474708 | 0.259653 | 0.171323 | 0.283981 |
| Range | 0.0362 | 0.0105 | 0.0348 | 0.0774 | 1.2188 | 0.2579 |
| Sum | 0.343 | 0.1337 | 0.1659 | 0.6426 | 15.5049 | 2.0411 |
| Sum of square | 0.02042 | 0.00307 | 0.005449 | 0.072689 | 41.04701 | 0.741011 |
| Geometoric mean | 0.05606 | 0.021967 | 0.025794 | 0.104621 | 2.551948 | 0.329457 |
| Harmonic mean | 0.055033 | 0.021677 | 0.024533 | 0.102609 | 2.519396 | 0.319605 |
| Skewness | 0.740617 | 0.732552 | 1.656452 | 1.542424 | -0.01728 | 0.632286 |
| Kurtosis | -0.45854 | -1.17814 | 0.972381 | 0.790175 | -1.16319 | -0.74553 |
| Median | 0.0551 | 0.0203 | 0.0239 | 0.09775 | 2.5563 | 0.33085 |
| Interquartile range | 0.01175 | 0.0052 | 0.00407 | 0.0088 | 0.524525 | 0.11185 |
| Mode | * | * | * | * | * | * |
| 10% Trimmed mean | 0.057167 | 0.022283 | 0.02765 | 0.1071 | 2.58415 | 0.340183 |
| Medianabsolute deviation | 0.0094 | 0.00295 | 0.006813 | 0.014867 | 0.35985 | 0.074917 |
|  |  |  |  |  |  |  |
| 1 CTRL |  |  |  |  |  |  |
|  | DHLNL | HLNL | PYD | Total  crosslinks | D/H | Maturation |
| Count | 6 | 6 | 6 | 6 | 6 | 6 |
| Mean | 0.020433 | 0.001817 | 0.002178 | 0.024417 | 11.36193 | 0.0959 |
| Unbiased Variance | 3.87E-05 | 1.58E-07 | 7.42E-07 | 5.22E-05 | 12.52016 | 0.000253 |
| Standard deviation | 0.00622 | 0.000397 | 0.000861 | 0.007223 | 3.538384 | 0.015894 |
| Standard error | 0.002539 | 0.000162 | 0.000352 | 0.002949 | 1.444539 | 0.006489 |
| Minimum | 0.0113 | 0.0013 | 0.00121 | 0.0138 | 8.6923 | 0.0697 |
| Maximum | 0.0291 | 0.0024 | 0.00332 | 0.034 | 18.1875 | 0.1145 |
| Coefficient of variation | 0.304382 | 0.218572 | 0.395474 | 0.295817 | 0.311425 | 0.165735 |
| Range | 0.0178 | 0.0011 | 0.00211 | 0.0202 | 9.4952 | 0.0448 |
| Sum | 0.1226 | 0.0109 | 0.01307 | 0.1465 | 68.1716 | 0.5754 |
| Sum of square | 0.002699 | 2.06E-05 | 3.22E-05 | 0.003838 | 837.162 | 0.056444 |
| Geometoric mean | 0.019564 | 0.001781 | 0.002027 | 0.023429 | 10.98761 | 0.094713 |
| Harmonic mean | 0.018629 | 0.001745 | 0.001876 | 0.022362 | 10.68983 | 0.093438 |
| Skewness | -0.15134 | 0.222415 | 0.03699 | -0.24336 | 1.412415 | -0.5598 |
| Kurtosis | -0.89508 | -1.0889 | -1.37719 | -1.01427 | 0.437478 | -0.67602 |
| Median | 0.0216 | 0.00175 | 0.0022 | 0.0258 | 9.78125 | 0.09775 |
| Interquartile range | 0.00615 | 0.00045 | 0.00128 | 0.007975 | 2.018 | 0.01625 |
| Mode | 0.0231 | 0.0016 | * | * | * | * |
| 10% Trimmed mean | 0.020433 | 0.001817 | 0.002178 | 0.024417 | 11.36193 | 0.0959 |
| Medianabsolute deviation | 0.004667 | 0.000317 | 0.000638 | 0.00545 | 2.0657 | 0.011467 |
|  |  |  |  |  |  |  |
|  |  |  |  |  |  |  |
| 2 TW tendon | |  |  |  |  |  |
|  | DHLNL | HLNL | PYD | Total  crosslinks | D/H | Maturation |
| Count | 6 | 6 | 6 | 6 | 6 | 6 |
| Mean | 0.0412 | 0.00955 | 0.009357 | 0.060083 | 4.455433 | 0.187733 |
| Unbiased Variance | 0.000111 | 4.91E-06 | 1.68E-06 | 0.000169 | 1.370267 | 0.000415 |
| Standard deviation | 0.010557 | 0.002216 | 0.001298 | 0.013008 | 1.170584 | 0.020362 |
| Standard error | 0.00431 | 0.000905 | 0.00053 | 0.005311 | 0.477889 | 0.008313 |
| Minimum | 0.0294 | 0.0059 | 0.00758 | 0.0477 | 2.7528 | 0.1606 |
| Maximum | 0.0589 | 0.0121 | 0.01141 | 0.0825 | 6.0816 | 0.2202 |
| Coefficient of variation | 0.256249 | 0.23205 | 0.138684 | 0.216503 | 0.262732 | 0.108463 |
| Range | 0.0295 | 0.0062 | 0.00383 | 0.0348 | 3.3288 | 0.0596 |
| Sum | 0.2472 | 0.0573 | 0.05614 | 0.3605 | 26.7326 | 1.1264 |
| Sum of square | 0.010742 | 0.000572 | 0.000534 | 0.022506 | 125.9567 | 0.213536 |
| Geometoric mean | 0.040152 | 0.009306 | 0.009283 | 0.059006 | 4.31828 | 0.186827 |
| Harmonic mean | 0.039192 | 0.009033 | 0.00921 | 0.058028 | 4.173861 | 0.185936 |
| Skewness | 0.720505 | -0.60435 | 0.325787 | 0.849955 | -0.12197 | 0.362865 |
| Kurtosis | -0.65966 | -0.73827 | -0.61125 | -0.57903 | -0.94237 | -0.65132 |
| Median | 0.03765 | 0.01005 | 0.00914 | 0.05615 | 4.5901 | 0.18495 |
| Interquartile range | 0.009925 | 0.002275 | 0.001043 | 0.013975 | 1.25105 | 0.01825 |
| Mode | 0.0358 | * | * | * | * | * |
| 10% Trimmed mean | 0.0412 | 0.00955 | 0.009357 | 0.060083 | 4.455433 | 0.187733 |
| Medianabsolute deviation | 0.007533 | 0.001683 | 0.000877 | 0.00965 | 0.8931 | 0.014933 |
|  |  |  |  |  |  |  |
|  |  |  |  |  |  |  |
| 3 TW tendon-like | |  |  |  |  |  |
|  | DHLNL | HLNL | PYD | Total  crosslinks | D/H | Maturation |
| Count | 6 | 6 | 6 | 6 | 6 | 6 |
| Mean | 0.02965 | 0.00665 | 0.004748 | 0.04105 | 4.571883 | 0.13155 |
| Unbiased Variance | 3.17E-05 | 2.35E-06 | 6.14E-07 | 5.27E-05 | 0.872704 | 0.000173 |
| Standard deviation | 0.005633 | 0.001532 | 0.000784 | 0.007257 | 0.934186 | 0.013171 |
| Standard error | 0.0023 | 0.000625 | 0.00032 | 0.002963 | 0.38138 | 0.005377 |
| Minimum | 0.0218 | 0.0051 | 0.00387 | 0.0324 | 3.2941 | 0.1155 |
| Maximum | 0.0387 | 0.009 | 0.00586 | 0.0525 | 5.6154 | 0.1523 |
| Coefficient of variation | 0.189984 | 0.230375 | 0.165052 | 0.176776 | 0.204333 | 0.100124 |
| Range | 0.0169 | 0.0039 | 0.00199 | 0.0201 | 2.3213 | 0.0368 |
| Sum | 0.1779 | 0.0399 | 0.02849 | 0.2463 | 27.4313 | 0.7893 |
| Sum of square | 0.005433 | 0.000277 | 0.000138 | 0.010374 | 129.7762 | 0.1047 |
| Geometoric mean | 0.029208 | 0.00651 | 0.004694 | 0.040537 | 4.487471 | 0.13101 |
| Harmonic mean | 0.02877 | 0.006379 | 0.00464 | 0.040048 | 4.399613 | 0.130481 |
| Skewness | 0.333595 | 0.516223 | 0.083382 | 0.546759 | -0.31883 | 0.39055 |
| Kurtosis | -0.47301 | -1.16086 | -1.27232 | -0.91672 | -1.40195 | -0.90865 |
| Median | 0.0286 | 0.0063 | 0.00484 | 0.03865 | 4.74695 | 0.13105 |
| Interquartile range | 0.0038 | 0.0021 | 0.001095 | 0.00725 | 1.3835 | 0.013975 |
| Mode | * | * | * | * | * | * |
| 10% Trimmed mean | 0.02965 | 0.00665 | 0.004748 | 0.04105 | 4.571883 | 0.13155 |
| Medianabsolute deviation | 0.003683 | 0.001183 | 0.000628 | 0.005083 | 0.72505 | 0.01045 |
